# The Pittsburgh Study: A Tiered Model to Support Parents during Early Childhood

**DOI:** 10.1016/j.jpeds.2024.114396

**Published:** 2024-11-12

**Authors:** Chelsea Weaver Krug, Alan L. Mendelsohn, Jordan Wuerth, Erin Roby, Daniel S. Shaw

**Affiliations:** 1 Department of Psychology, University of Pittsburgh, Pittsburgh, PA; 2 NYU Grossman School of Medicine, New York, NY

## Abstract

**Objective:**

To test the feasibility of implementing The Pittsburgh Study’s (TPS) Early Childhood Collaborative, a population-level, community-partnered initiative to promote relational health by offering accessible preventive parenting program options for families with young children.

**Study design:**

TPS partnered with healthcare and community agencies serving families in Allegheny County, Pennsylvania, to enroll and screen 878 parents of 1040 children 4-years-old and under. Participants were assigned to 1 of 4 tiered groups based on identified needs: (1) universal, (2) targeted/universal, (3) secondary/tertiary, or (4) tertiary programs. Parents were offered choices in empirically supported parenting programs within group ranging from texting programs to intensive home visiting. Program selection was optional. Chi-square tests were conducted to examine the likelihood of selecting a program by group.

**Results:**

About 25% of participants were assigned to each tiered group; 78% of parents chose to enroll in a parenting program. In general, parents with higher levels of adversity were more likely to select a parenting program compared with those reporting less adversity, including secondary/tertiary vs targeted/universal groups (81.4% vs 72.8%), and tertiary vs universal and targeted/universal groups (83% vs 74.1% and 72.8%, respectively; *P* < .001).

**Conclusions:**

Our high program enrollment rate supports the feasibility of TPS. TPS successfully engaged families in the study by offering choices in, and optimizing accessibility to, parenting programs. TPS is highly aligned with recent recommendations by the American Academy of Pediatrics for tiered approaches as part of a broad public health strategy for supporting early relational health. *(J Pediatr 2025;277:114396)*.

**Trial Registration:**

The Pittsburgh Study Early Childhood (TPS-ECC): NCT05444205.

Chronic adversity during early childhood is a pervasive public health problem and contributes to health inequities and childhood toxic stress.^1^ Factors associated with chronic adversity include structural racism and economic marginalization, such as living in dangerous neighborhoods with concentrated poverty and experiencing discrimination.^2^ In addition, extreme adversity (eg, abuse, homelessness) may place children at high risk for toxic stress, which may persist into adulthood.^3^ Parent-child relational health may buffer the effects of adversity and prevent toxic stress.^4^

Empirically supported parenting programs have been shown to improve relational health for families with young children in poverty.^5^ However, identification of families and parent accessibility to services persist as barriers to engagement.^6^ Furthermore, community-based approaches to improve health inequities have shown promising results.^2,7^ The American Academy of Pediatrics (AAP) published recommendations in 2021 for a public health, community-partnered approach to promote relational health and prevent childhood toxic stress at population-level.^4^ The feasibility of such an approach with integrated implementation across both the health care system and the community has not yet been fully studied.

The Pittsburgh Study’s Early Childhood Collaborative (TPS) is a novel population-level public health approach for bolstering early child development via promoting relational health with supportive parenting programs. The innovative design of TPS, which is aligned with AAP recommendations, utilizes strategies including the following: 1) partnerships with public service systems and 2) supporting parent choice from a menu of universal, secondary, and tertiary prevention programs tailored to family-identified strengths and challenges (see Figure 1). TPS addresses 4 barriers that have limited impacts at the individual and population level: 1) identifying and engaging families historically marginalized based on race and income; 2) scaling up at low cost by leveraging existing public service systems; 3) offering services based on risk-stratified, tiered groups to address heterogeneity in barriers to relational health among families (eg, poverty, mental health challenges, child welfare involvement); and 4) optimizing accessibility and reducing cost, by offering programs remotely and at locations families already frequent, such as pediatric clinics, Supplemental Nutrition Program for Women, Infants, and Children (WIC), libraries, and family residences.

The current report tests the feasibility of TPS as a public health approach closely aligned with the AAP pyramid approach to prevent child toxic stress and promote relational health^4^ by (1) examining the proportion of participants assigned to 4 tiered groups and (2) program selection rates across and within groups.

## Methods

### Community Partnerships

An important component of the study design was establishing meaningful partnerships with healthcare and community agencies serving families with young children, particularly families historically marginalized based on race and income. Supplemental sTable 1 online; available at www.jpeds.com contains details about the collaborations built with community service providers, which formed the foundation of TPS’ horizontal approach to study recruitment, assessment, and program delivery.

Further, a Community Collaborative comprised of residents primarily from marginalized neighborhoods was convened to partner with TPS to develop study materials (eg, flyers, consent forms, newsletters) to ensure that study methods were aligned with the priorities of the communities TPS served. The Community Collaborative members were initially engaged primarily via Family Centers, were trained in human subjects research, and participated in regular meetings for which they were compensated.

### Study Enrollment

TPS recruitment occurred remotely and in person in Allegheny County, Pennsylvania, at several locations including a birthing hospital that accounts for 45% of all births in the county (more than 10 000 annually), Allegheny County WIC clinic locations serving approximately 16 000 families, and a pediatric clinic system serving over 5000 primarily low-income young children annually. Primary parent (P1)-target child dyads were eligible to participate if P1 was the legal custodian and primary caretaker of a child under 4 years old (to allow time for program participation), resided in Allegheny County, and were fluent in English.

A second parent (P2) could enroll in the study at the discretion of P1. All P1s were asked if there was someone who helped parent their child who may be interested in participating with them. P2s had similar eligibility criteria except for legal custody to include families with diverse caregiver structures (eg, grandparents, father-figures). P2s were either co-enrolled with P1s or were contacted via phone and enrolled remotely. Further, families could enroll multiple children under 4 years old. Overall, 80% of P1-child dyads screened for eligibility enrolled in the study; 95% of screened P2s enrolled (see Supplemental sFigure 1, online; available at www.jpeds.com). Enrollment of the current sample occurred June 2020 to September 2023. TPS recruitment was ongoing at the time of this report. Study procedures were approved by the local institutional review board.

### Measurement and Tiered Groups

Participants completed 20–25-minute surveys on study iPads or via online links to assess strengths, adversity, and risk for toxic stress. Specifically, sociodemographics (eg, income, teen parent status, homelessness), child health risk and well-being (eg, preterm birth, neonatal intensive care, injuries, difficultness, problem behavior), supportive/proactive parenting, parent well-being (eg, depressive/anxiety symptoms, opioid use), social support, system involvement (child welfare, incarceration) were assessed (see Table I for measure descriptions and scoring procedures). Assessment constructs were selected based on (1) established effects of the evidence-based programs that were offered on relational health and child psychosocial outcomes (described in Supplemental sTable 2 online; available at www.jpeds.com) and (2) the severity of risk for toxic stress for the parent or child. Assessments were scored in real time using procedures in Table I to assign parents to 1 of 4 groups characterized by the type of programs offered: Group 1: Universal Programs (G1-Univ), Group 2: Targeted/Universal Programs (G2-TargetUniv), Group 3: Secondary/Tertiary Programs (G3-SecTer), and Group 4: Tertiary Programs (G4-Tertiary). Specifically, participants were assigned to the highest group for which they met any eligibility criteria outlined in Table I. For example, a parent who met low-income criterion and no other measured adversity would be assigned to G2-TargetUniv. A parent with elevated depressive symptoms would be assigned to G3-SecTer if no criteria for G4-Tertiary were met. A parent who endorsed opioid use would be assigned to G4-Tertiary regardless of other reported adversity.

### Program Offerings

TPS sought to match services to identified family needs and risks, with approximate alignment with the AAP prevention pyramid recommended as a public health strategy for population-level prevention.^4^ For example, TPS offered universal texting programs to families who endorsed no measured risk factors (G1-Univ), which corresponds to level 1 of the prevention pyramid (primary prevention). Next, G2-TargetUniv included families reporting economic challenges but limited psychosocial adversities, and thus were offered targeted universal programs of greater intensity than G1-Univ, but nonetheless applicable to families in level 1 of the prevention pyramid. Such programs included PlayReadVIP, which has demonstrated positive outcomes for families with low incomes facing variation in psychosocial adversities (see Supplemental sTable 2, online; available at www.jpeds.com). G3-SecTer was designed to bridge levels 2 and 3 (secondary and tertiary) of the prevention pyramid. As such, participants in G3-SecTer were offered more intensive targeted programs to address, and/or repair relational health, including Family Check-Up (FCU), which has been shown to be more efficacious in improving child behavior for low-income families facing multiple psychosocial adversities (see Supplemental sTable 2, online; available at www.jpeds.com). Finally, parents reporting the highest levels of adversity and likely toxic stress (eg, homelessness, child welfare involvement), were offered the most intensive home visiting programs as part of G4-Tertiary. Supplemental sTable 2, online; available at www.jpeds.com contains descriptions of the full menu of programs by group.

Each program option was described in a standardized manner to parents either in person, via phone, or via telehealth. In addition, 1–3-minute videos developed by Nurture Program, PlayReadVIP, and FCU about their respective programs were shared with participants when appropriate. Parents were then invited to choose the program(s) that best fit their family’s needs, or to decline programs while remaining in the study. If P1-P2 dyads planned to participate in programs together, a family-centered approach was used by offering programs to the family unit based on the parent assigned to the highest need group. P1-P2 dyads who did not wish to participate together were offered programs separately based on each parent’s respective group assignment. If participants were dissatisfied with their options and asked for alternative programs, they were offered programs decreasing in intensity. For example, if a participant in G3-SecTer was not interested in PlayReadVIP or FCU and asked if there was a less time-consuming option, they would be offered the programs for G2-TargetUniv. Programs more intensive than those presented were not offered, as it was not economically viable.

Moreover, because population segmentation could vary based on child factors, participants with multiple children enrolled were offered programs associated with the group assignment for each child. For example, if a P1 reported few challenges with their infant and a toddler exhibiting significant conduct problems, TPS would offer lesser intensive programs related to the infant and targeted programs focused on relational health with the toddler. If intensive programs were indicated across children, families were encouraged to decide on a single program that would best serve their family in an integrated way. At follow-up assessments, participants could continue, add, or remove programs.

Lastly, each family was assigned to a Clinical Navigator for the duration of the study. Navigators were staff with clinical experience serving families and expertise in services available to residents of Allegheny County. Navigators provided individualized support for program selection and also worked with families to obtain local resources (eg, food pantries, diaper banks), creating a layered approach to prevention services.

### Statistical Analysis

Statistical analyses were conducted to describe indicators of feasibility; specifically, proportions of families assigned to each group and the degree to which families in each group chose to participate in a program. Differences in groups based on sociodemographic variables and comparisons of program selection based on group membership were analyzed in SPSS using likelihood-ratio chi-square tests.

## Results

Participants were 878 P1s and 1045 target children. P1s were mostly biological mothers (96.4%); 53.1% were White and 51.2% had an annual household income less than $30,000 (average between $48,012 and $54,000). Sample demographics were not representative of Allegheny County due to oversampling of low-income families. According to the US Census Bureau, about 75.7% of Allegheny County residents were White in 2020 and the median household income of residents was $76,615 in 2023, with about 12.5% of residents living in poverty^16^ (ie, below $30,000 per year for a family of 4). Of enrolled P1s, 187 (21.3%) enrolled a P2. P2s were mostly biological fathers (88.2%); 66.8% were White. Families with an enrolled P2 had an average annual household income between $78,012 and $84,000, with 28.4% earning less than $30,000.

Of the 878 P1s, 124 (14.1%) enrolled 2 children and 20 (2.3%) enrolled 3 children in the study. Child age at enrollment ranged from 0 to 53 months (average age = 8 months); 35.8% of children were enrolled before 2 weeks of age. Results of tiered grouping showed that the proportion of P1s assigned to each group ranged from 23.0% assigned to G2-TargetUniv to 29.4% G3-SecTer whereas P2 proportions ranged from 17.1% assigned to G2-TargetUniv to 36.4% assigned to G1-Univ. P1 and P2 demographics by group are shown in Table II.

In terms of barriers to relational health that determined group assignment, annual family income under $30,000 was the most common qualifying factor for P1s and P2s (96.7% and 52.2%, respectively) assigned to G2-TargetUniv. In G3-SecTer, mental health problems (eg, elevated depression or anxiety symptoms) were the most common qualifiers for P1 and P2 (81.5% and 89.7%, respectively). The most common criterion of G4-Tertiary membership was child welfare involvement for P1s (60.5%) and prior incarceration for P2s (66.7%). Supplemental sTable 3, online; available at www.jpeds.com contains qualifying criteria by group for P1s and P2s.

Overall, 78.0% of P1s chose to participate in a parenting program. P1s in G3-SecTer were significantly more likely to select a program compared with parents in G2-TargetUniv (81.4% vs 72.8%); importantly, P1s in G4-Tertiary were significantly more likely to choose a program than those in G1-Univ and G2-TargetUniv (83% vs 74.1% and 72.8%, respectively; *P* < .001). Comparatively, 73.5% of P2s chose to participate in a parenting program. Program selections by program group are presented in Supplemental sTable 4, online; available at www.jpeds.com. Note that 21.3% of parents in G4-Tertiary were offered a choice between Smart Beginnings and Healthy Families America because they had eligible newborns; otherwise, parents in G4-Tertiary were offered the choice between Smart Beginnings and FCU.

Only 11.8% of P1s and 16.7% of P2s declined to participate in a parenting program. P1s who declined programs were less likely to receive WIC (*P* < .001), more likely to be married (*P* < .001), less likely to identify as Black (*P* < .05), and less likely to have an enrolled P2 (*P* < .05). Similar patterns were found for P2s who declined programs. There were no significant differences between P1s or P2s who declined vs selected programs in terms of parent gender, employment status, or Latinx identity. Lastly, 9% of P1s and 6.5% of P2s were unable to be contacted to provide program options after completing the survey.

## Discussion

Our findings support a core component of the feasibility of TPS as a model for supporting relational health and preventing childhood toxic stress. Specifically, a tiered model was used to offer universal, secondary, and tertiary prevention program options to families and acceptability was supported, with 78% of families choosing to participate in a parenting program. As TPS’ design is aligned with AAP recommendations for preventing toxic stress via promoting relational health, TPS’ strategy of screening, using a tiered approach, giving families’ choices of programs within tiers, and making these programs accessible may provide a pathway for actualizing AAP’s public health strategy.^4^

TPS brought together several innovative strategies to promote study engagement. First, TPS’ approach leveraged established relationships that parents had with trusted community providers (eg, pediatric clinics, WIC offices), which not only served as the foundation for identification of families with young children who have been historically marginalized, but also promoted accessibility for parents by offering parenting programs at locations participants frequented. In addition, in-home and virtual service delivery options were offered to optimize engagement, addressing common barriers, such as busy schedules and transportation challenges.

Importantly, TPS strategies were especially successful in engaging families who were offered the most intensive programs, compared with families with lower identified program needs. This finding is especially important as families experiencing significant adversity are often the most difficult to engage and retain in clinical services.^17,18^

Relatedly, TPS offered participants choices of parenting programs tailored to self-identified strengths and challenges, including the choice to decline programs while remaining in the study. TPS’s strength-based approach^19^ of providing options was designed to demonstrate respect while promoting agency and intrinsic motivation to select a program. It must be considered that the tailored program options did not include the full menu of programs, thus limiting their options. However, offering intensive services to families who reported many resources and few challenges would not be economically viable. Moreover, offering light-touch programs to families facing extreme adversity may do them a disservice by not adequately addressing their needs, potentially overlooking accessible and effective programs tailored for families in similar circumstances. Nonetheless, study results showed that families largely selected programs that aligned with their needs based on tier-based group assignment.

The current study had several limitations. First, risk stratification was based on self-reports, relying on participants to disclose adverse experiences. Future research could consider using healthcare records to identify program needs and provide more objective estimates, especially for parents experiencing toxic stress who may be reticent to divulge their challenges. Supplementing the current approach with official records likely would have identified more parents most in need of intensive services; however, this strategy could have its downsides, such as circumventing the opportunity to build trust with participants; a core component of this work.

Next, TPS was implemented within largely urban and suburban Allegheny County limiting generalizability to other racial or ethnic groups and geographic locations. For example, less than 5% of the current sample identified as Hispanic/Latinx and it is unknown whether risk stratification or program selections would differ for racial and ethnic groups who were not well-represented in TPS. Furthermore, parents in rural communities may face unique obstacles around accessibility, such as lack of transportation or internet access,^20^ which would present barriers to engaging participants in online surveys and remote program delivery.

Study recruitment at birthing hospitals, WIC, and pediatric clinics resulted in the majority of P1s being biological mothers. Despite asking all P1s if a co-parent would like to enroll in TPS, biological fathers engaged in less than one-fifth of cases. Interestingly, recruitment rates for fathers were relatively higher at birthing hospitals (75% of P2s vs 59% of P1s). Future research should improve strategies to enhance father engagement, such as offering father-specific program content/options.

Lastly, while TPS strategies were effective in motivating parents to select parenting programs, initial and sustained program engagement were not evaluated; this will become available in the coming years. Evidence suggests that greater participation in parenting programs yields more robust effects even after accounting for level of adversity.^21,22^ Future research should evaluate levels of program engagement over time as barriers to relational health may evolve, especially among families facing significant adversity. Of additional relevance, the programs most often selected by families indicated for targeted prevention programs (ie, FCU, PlayReadVIP) have been shown to have high levels of engagement individually and potentiate engagement in each other when delivered as part of the integrated SB model.^6^

In conclusion, TPS demonstrated several important methods that successfully engaged parents in the study, including offering choices between targeted, empirically supported primary, secondary, and tertiary parenting programs. Based on the alignment between TPS and AAP recommendations for a public health approach to preventing toxic stress,^4^ the findings provide initial support for the feasibility of implementing AAP’s population-level strategy. The structure and framework of TPS may be adapted for implementation in other communities by tailoring partnerships, screening assessments, and programs to fit the specific needs of each population.

Future directions include examining program engagement rates in selected programs and testing program impacts on relational health and child psychosocial outcomes in the context of a population-level implementation. The current study’s findings can inform prevention science broadly and support the notion that population-level prevention models may benefit from establishing collaborations between pediatric health care and community settings to leverage relationships that parents have with trusted providers.

## Supplementary Material

JPeds 2025 supplementary materials

## Figures and Tables

**Figure 1.**
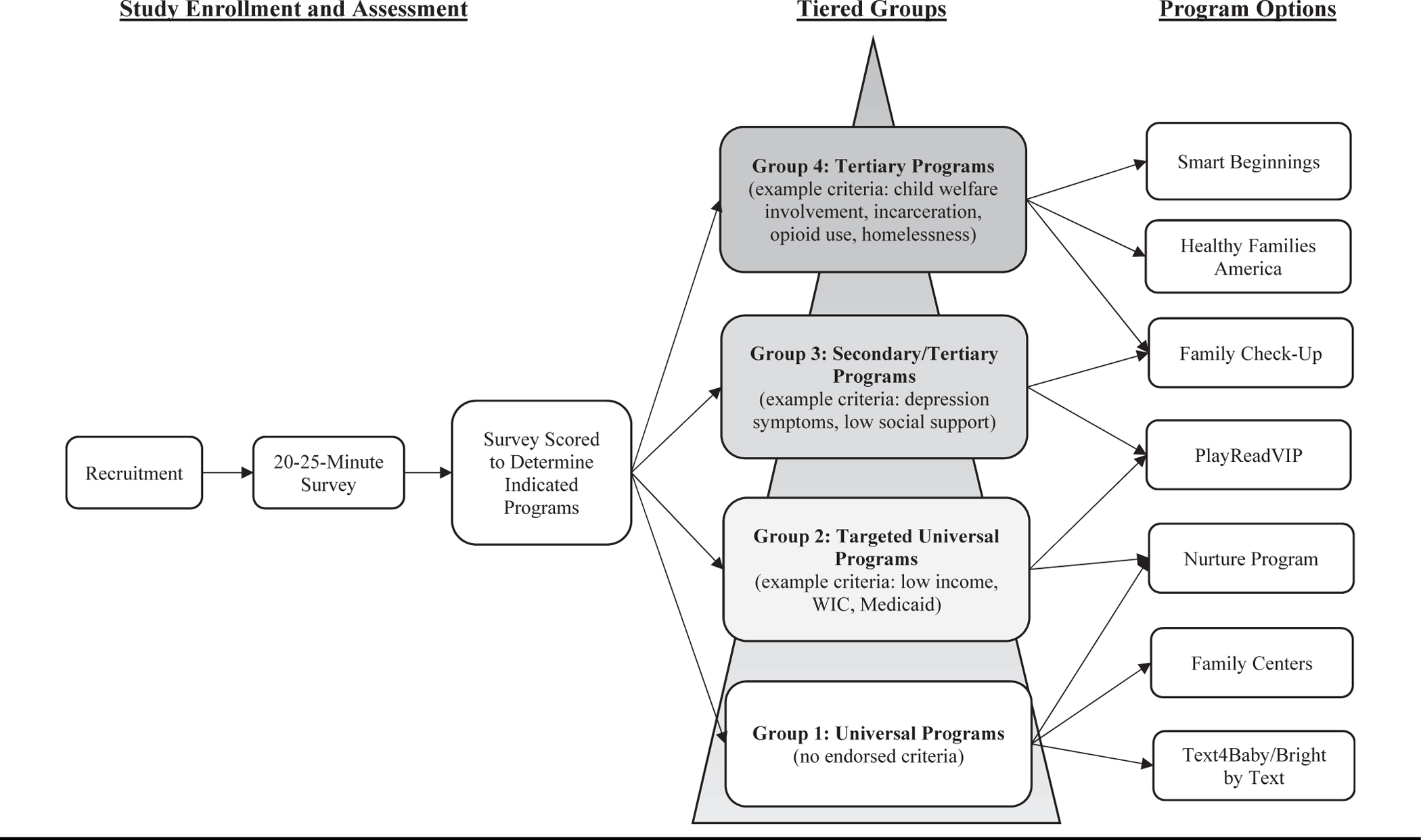
Tier-based approach to providing supportive parenting program options to parents with young children.

**Table I.** Measures used to determine tier-based groups

Measures by group assignment	Description	Scoring procedure

Group 2: Universal		
Low-income	Self-reported categories of monthly/annual income ranges and additional sources of financial support.	Annual household income <$30k*, WIC receipt, or Medicaid receipt.
Teen parent	Calculated using parent date of birth and date of study enrollment.	Parent age < 20 years at enrollment
Birth complications	Two items: (1) Was this child born at less than 37 weeks gestation; that is, was s/he more than 3 weeks early? And (2) Did your baby spend 4 or more nights in the NICU?	Dichotomous endorsement of either item.
Does not read with child: Stim-Q2 (Infant, Toddler, Preschool versions)^8^	The reading subscale was administered. Items used for risk stratification: (1) Do you get to read baby or children’s books to your baby or is your child too young for that?	Dichotomous endorsement of No, my child is still too young to read books together.
Mild parenting challenges: PYB/PYT^9^	13-item measure of parenting behavior frequency in the domains of supportive and proactive parenting. Response scale ranged from 1 (Not at all) to 7 (Most of the time). Items contained on PYB vs PYT varied to be developmentally appropriate. PYB was administered if the child was between 3 and 18 months old; PYT was administered between 18 and 36 months old.	Mean scores <6 and >3 for supportive and proactive parenting†.
Group 3: Secondary/Tertiary		
Mental health problems: Hospitalization history	Single item: Were you ever hospitalized for a mental health problem; that is, you stayed overnight at a hospital or other mental health treatment facility?	Dichotomous endorsement.
Mental health problems: Center for Epidemiologic Studies on Depression Scale^10^	20-item self-report measure of depressive symptoms. Response scale ranged from 0 (less than 1 day in the past week) to 3 (5–7 days in the past week).	Sum scores > 12
Mental health problems: Generalized Anxiety Disorder – 7^11^	7-item self-report measure of anxiety symptoms in the past 2 weeks. Response scale ranged from 0 (not at all) to 3 (nearly every day).	Sum scores > 9
Moderate parenting challenges: PYB/PYT^9^	Measure details reported under Group 2 Universal – Moderate Intensity qualifiers	Mean scores≤3 for supportive and proactive parenting^1^.
Low social support: Comprehensive Inventory of Thriving^12^	3-item Support subscale. Response scale ranged from 1 (strongly disagree) to 5 (strongly agree).	Sum scores < 7
Child injuries	Single item: Since your child began living with you/came home after birth, how many times has he/she seen a doctor or other medical professional or visited a clinic or emergency room for an injury? Administered if child was > 6 months old.	Responses of 3 or more times.
Child Difficultness: Infant Characteristics Questionnaire^13^	7-item Difficultness factor assessed characteristics and behaviors that were typical for their infant. Response scale ranged from 1 to 7 with anchors varying throughout (eg, Very easy to Difficult and Never to More than 15 times per day). Administered if child was between 24 and 36 months old.	Sum scores≥24
Group 4: Tertiary		
Recent homelessness^14^	Assessed using 2 items: (1) What is your current type of housing? and (2) Have you been homeless at any time in the past 2 years? By homeless, we mean that you were not living in stable housing that you own, rent, or stay in as part of a household.	Type of housing = Temporary housing (eg, women’s shelter, homeless shelter) or if current homelessness or homelessness in the past 2 years.
Service involvement	Items used for risk stratification: Have you ever been contacted by child protective services or CYF (Children Youth and Family Services) about a child who was/is in your care? and (2) Have you ever been incarcerated in jail or prison?	Dichotomous endorsement of either service.
Opioid use	Dichotomous assessment of the use of 10 types of substances during the last 6 months.	Endorsements of Opioids (eg, heroin, fentanyl, oxycodone, etc.) without a prescription or other than prescribed (using more or more often) or Buprenorphine (eg, Cizdol, Suboxone, Subutex) or Methadone (eg, Dolophine, Methadose)
Child problem behavior: Brief Infant Toddler Social Emotional Assessment^15^	37-item problem behavior subscale assessed internalizing and externalizing behaviors. Response scale ranged from 0 (Not True/Rarely) to 2 (Very True/Often). Administered if child was between 24 and 36 months old.	The instrument’s clinical cutoffs based on child sex and age at assessment were used.

*NICU,* neonatal intensive care unit; *PYB,* Parenting Your Baby, *PYT,* Parenting Your Toddler.

Participants who did not endorse any measured factors used for tier-based grouping were assigned to Group 1: Universal.

* Poverty line for a family of 4 in Allegheny County in 2023.

† Scoring cutoffs for PYB/PYT were informed by published descriptive statistics^9^ and expertise of clinicians who used the PYB and PYT in their work with families in Allegheny County.

**Table II.** Parent 1 (P1) and Parent 2 (P1) demographics by group

	P1 (n = 878)				
	Group 1: Universal n (% sample)	Group 2: Targeted universal n (% sample)	Group 3: Secondary/tertiary n (% sample)	Group 4: Tertiary n (% sample)	
P1 group proportions	212 (24.1%) n (% group)	202 (23.0%) n (% group)	258 (29.4%) n (% group)	206 (23.5%) n (% group)	Chi-square (df)

Demographic variables*					
Relationship to child^†^					
Biological mother	206 (97.2%)	192 (95.0%)	252 (97.7%)	196 (95.1%)	*X^2^*(3) = 2.53, *ns*
Biological father	6 (2.8%)	7 (3.5%)	5 (1.9%)	4 (1.9%)	NA
Grandparent	0 (0%)	1 (0.5%)	1 (0.4%)	1 (0.5%)	NA
Other (eg, adoptive parent, parent’s partner)	0 (0%)	2 (1.0%)	0 (0%)	5 (2.4%)	NA
Gender					
Female	202 (95.3%)	187 (92.3%)	241 (93.4%)	186 (90.3%)	*X^2^*(3) = 4.10, *ns*
Male	10 (4.7%)	15 (7.4%)	14 (5.4%)	19 (9.2%)	*X^2^*(3) = 4.32, *ns*
Nonbinary/third gender	0 (0%)	0 (0%)	3 (1.2%)	0 (0%)	NA
Hispanic/Latinx ethnicity	5 (2.4%)	9 (4.4%)	8 (3.1%)	7 (3.4%)	*X^2^*(3) = 1.47, *ns*
Race					
Black	21 (10.0%)^a^	128 (63.4%)^b^	117 (45.3%)^c^	132 (64.1%)^b^	*X^2^*(3) = 163.08, *P* <.001
White	171 (80.7%)^a^	58 (28.7%)^b^	121 (46.9%)^c^	64 (31.1%)^b^	*X^2^*(3) = 144.48, *P* <.001
Other races	19 (8.9%)	16 (7.9%)	20 (7.8%)	10 (4.8%)	*X^2^*(3) = 2.83, *ns*
Marital status					
Married or living with partner	196 (92.5%)^a^	78 (38.6%)^b^	142 (55.0%)^c^	52 (25.2%)^d^	*X^2^*(3) = 306.51, *P* <.001
Single	13 (6.1%)^a^	120 (59.4%)^b^	108 (42.0%)^c^	142 (68.9%)^d^	*X^2^*(3) = 195.60, *P* <.001
Divorced, separated, or widowed	0 (0%)	4 (2.0%)	8 (3.1%)	10 (4.8%)	NA
Employed	180 (85.0%)^a^	85 (42.1%)^b^	155 (60.1%)^c^	88 (42.7%)^b^	*X^2^*(3) = 105.18, *P* <.001
Education					
Partial high school or less	1 (0.5%)	5 (2.5%)	10 (3.9%)	15 (7.2%)	NA
High school or GED	8 (3.8%)^a^	72 (35.6%)^b^	60 (23.2%)^c^	75 (36.4%)^b^	*X^2^*(3) = 78.83, *P* < .001
Partial college, associate’s degree, or specialized Certification	19 (9.0%)^a^	74 (36.6%)^b^	76 (29.5%)^b^	73 (35.4%)^b^	*X^2^*(3) = 51.92, *P* < .001
College graduate	70 (33.0%)^a^	20 (9.9%)^b^	43 (16.7%)^c^	11 (5.3%)^b^	*X^2^*(3) = 67.32, *P* < .001
Graduate school or graduate degree	114 (53.8%)^a^	31 (15.3%)^b^	69 (26.7%)^c^	31 (15.0%)^b^	*X^2^*(3) = 103.42, *P* <.001
Monthly household income					
Less than $30,000/year	7 (3.3%)^a^	148 (73.6%)^b^	131 (50.8%)^c^	168 (81.6%)^d^	*X^2^*(3) = 310.11, *P* <.001
$30,000–$60,000/year	35 (16.5%)	39 (19.3%)	46 (17.8%)	29 (14.1%)	*X^2^*(3) = 2.17, *ns*
$60,000–$90,000/year	35 (16.5%)	10 (5.0%)	31 (12.0%)	4 (1.9%)	NA
More than $90,000/year	132 (62.3%)	5 (2.5%)	47 (18.2%)	2 (1.0%)	NA

	P2 (n = 187)				
	Group 1: Universal n (% sample)	Group 2: Targeted universal n (% sample)	Group 3: Secondary/tertiary n (% sample)	Group 4: Tertiary n (% sample)	
P2 group proportions	68 (36.4%) n (% group)	32 (17.1%) n (% group)	51 (27.3%) n (% group)	36 (19.3%) n (% group)	Chi-square (df)

Demographic variables*					
Relationship to child^†^					
Biological mother	2 (2.9%)	2 (6.3%)	3 (5.9%)	3 (8.3%)	NA
Biological father	66 (97.0%)^a^	25 (78.1%)^b^	40 (78.4%)^b^	31 (86.1%)^b^	*X^2^*(3) = 11.35, *P* <.05
Grandparent	0 (0%)	1 (3.1%)	8 (15.7%)	1 (2.8%)	NA
Other (eg, adoptive parent, parent’s partner)	0 (0%)	4 (12.5%)	0 (0%)	1 (2.8%)	NA
Gender I					
Female	2 (2.9%)	5 (15.6%)	12 (23.5%)	5 (13.9%)	NA
Male	66 (97.0%)^a^	27 (84.4%)^b^	39 (76.5%)^b^	30 (83.3%)^b^	*X^2^*(3) = 11.41, *P* <.05
Nonbinary/third gender	0 (0%)	0 (0%)	0 (0%)	0 (0%)	NA
Hispanic/Latinx Ethnicity	0 (0%)	1 (3.1%)	1 (2.0%)	1 (2.8%)	NA
Race					
Black	8 (11.8%)^a^	16 (50.0%)^b c^	15 (29.4%)^c^	19 (52.8%)^b^	*X^2^*(3) = 25.20, *P* < .001
White	60 (88.2%)^a^	15 (46.9%)^b^	29 (56.9%)^b^	15 (41.7%)^b^	*X^2^*(3) = 30.19, *P* < .001
Other Races	0 (0%)	1 (3.1%)	7 (13.7%)	2 (5.5%)	NA
Marital status					
Married or living with partner	66 (97.0%)^a^	20 (62.5%)^b^	40 (78.4%)^c^	24 (66.7%)^b^	*X^2^*(3) = 32.87, *P* < .001
Single	1 (1.5%)	12 (37.5%)	10 (19.6%)	12 (33.3%)	NA
Divorced, separated, or widowed	1 (1.5%)	0 (0%)	1 (2.0%)	0 (0%)	NA
Employed	68 (100.0%)^a^	28 (87.5%)^b^	38 (74.5%)^b^	17 (47.2%)^c^	*X^2^*(3) = 44.46, *P* < .001
Education					
Partial high school or less	0 (0%)	0 (0%)	2 (3.9%)	2 (5.5%)	NA
High school or GED	4 (5.9%)	11 (34.4%)	7 (13.7%)	12 (33.3%)	NA
Partial college, associate’s degree, or specialized Certification	0 (0%)	0 (0%)	2 (3.9%)	2 (5.5%)	NA
College graduate	29 (42.6%)	3 (9.4%)	9 (17.6%)	0 (0%)	NA
Graduate school or graduate degree	28 (41.2%)	11 (34.4%)	21 (41.2%)	7 (19.4%)	*X^2^*(3) = 5.71, *ns*
Monthly household income					
Less than $30,000/year	0 (0%)	18 (56.3%)	14 (27.5%)	22 (61.1%)	NA
$30,000–$60,000/year	13 (19.1%)	9 (28.1%)	10 (19.6%)	5 (13.9%)	NA
$60,000–$90,000/year	10 (14.7%)	3 (9.4%)	4 (7.8%)	5 (13.9%)	NA
More than $90,000/year	45 (66.2%)	2 (6.3%)	20 (39.2%)	1 (2.8%)	NA

Notes: chi-square tests were performed only if all groups had n > 5 participants. Superscript letters for significant test results denote tier-based groups whose proportions do not differ significantly from each other at the .05 level.

* Demographic information is reported for each unique parent participant. Note that a subset of parents had multiple children enrolled in the study.

† Relationship to Child references the first enrolled child if the parent had multiple children enrolled in the study.

## Data Availability

Data sharing statement available at www.jpeds.com.

## Abbreviations

TPS: The Pittsburgh Study
AAP: American Academy of Pediatrics
WIC: Supplemental Nutrition Program for Women, Infants, and Children
P1: Primary Parent
P2: Second Parent
G1-Univ: Group 1 Universal Programs
G2-TargetUniv: Group 2 Targeted/Universal Programs
G3-SecTer: Group 3 Secondary/Tertiary Programs
G4-Tertiary: Group 4 Tertiary Programs
FCU: Family Check-Up
