# Supplementary material for: The Pittsburgh Study: A Tiered Model to Support Parents during Early Childhood
Source: J Pediatr. Author manuscript; Available in PMC 2025 Aug 8. (PMC12333661; doi:10.1016/j.jpeds.2024.114396)
Supplement: JPeds 2025 supplementary materials [file NIHMS2099469-supplement-JPeds_2025_supplementary_materials.pdf]

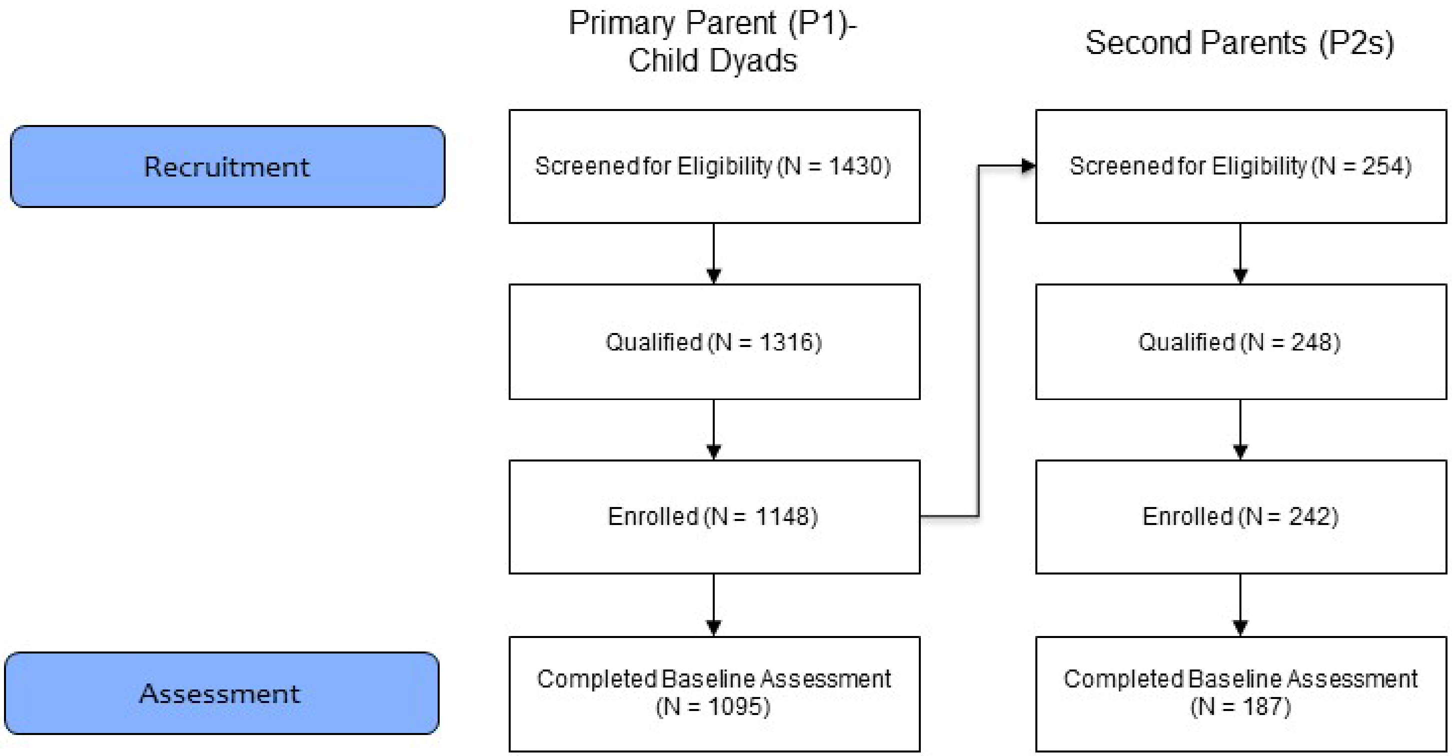
Primary Parent (P1)-
Child Dyads
Second Parents (P2s)
Recruitment
Screened for Eligibility (N = 1430)
Screened for Eligibility (N = 254)
Qualified (N = 1316)
Qualified (N = 248)
Enrolled (N = 1148)
Enrolled (N = 242)
Assessment
Completed Baseline Assessment
(N = 1095)
Completed Baseline Assessment
(N = 187)

sTable 1. Community partnerships and collaboration activities.

Agency	Locations	Study Activity	Collaboration
University of Pittsburgh General Academic Pediatrics	Two neighborhood pediatric clinics	Study recruitment and program delivery	Meetings at pediatric clinic sites to develop recruitment process and locate space to deliver PlayReadVIP (following implementation of PlayReadVIP and Family Check-Up during a National Institutes of Health-funded trial).<br><br>Study investigators delivered presentations about the study to clinic staff (physicians, nurses, and administrative personnel).
Allegheny County Department of Human Services	Family Centers	Study recruitment, program delivery, and referrals to Family Center programs	Regular meetings to develop a streamlined referral process and data sharing agreements to evaluate program impacts (following implementation of Family Check-Up during a state-funded trial).<br><br>Collaborated to find strategies to avoid duplication of services.<br><br>Assistant Deputy Director joined the study investigator team and attended regular research meetings.<br><br>Study investigators delivered presentations about the study to Family Center staff (leadership and Family Development Specialists).
Allegheny County Department of Health	Allegheny County Supplemental Nutrition Program for Women, Infants, and Children (WIC) Nutritional Program	Study recruitment and program delivery	Meetings at WIC sites and clinic tours to develop recruitment process and locate space to deliver PlayReadVIP (following NIH trial to deliver Family Check-Up).<br><br>Study investigators delivered presentations about the study to clinic staff (leadership, nutritionists, and administrative personnel).<br><br>Developed strategy with WIC Program Manager (and obtained state approval) to send text messages of families to assess interest in the study during the Covid-19 pandemic.
Allegheny County Health Department	Office of Family and Child Health	Study referrals to Healthy Families America	Regular meetings with Family and Child Health Program Manager to develop a streamlined referral process and data sharing agreements to evaluate program impacts.<br><br>Family and Child Health Program Manager joined study Investigator committee and attended regular research meetings.

University of Pittsburgh Medical Center	Birthing Hospital	Study recruitment	Meetings to develop and maintain recruitment process on the mother-baby unit after delivery.
	Local neighborhood clinics	Study recruitment and program delivery	
Nurture Program	NA	Study referrals to Nurture Program	Regular meetings to develop a streamlined referral process and data sharing agreements to evaluate program impacts.<br><br>As needed meetings to maintain effective referral processes.

sTable 2. Parenting program menu, empirical evidence, and referral processes

Program Name	Program Type	Program Description	Empirical Evidence	Groups Offered	Referral Process
Text4baby	Universal	Text messaging program designed to target health inequities of mothers in poverty[16]. Text4baby promotes healthy pregnancies and babies in the first year of life through developmentally appropriate text messages.	A Health Resources and Services Administration-funded evaluation found significantly improved knowledge of safe sleep and infant feeding among mothers enrolled in Text4baby[17]	Group 1: Universal (when child was under 9 months to provide time for program participation)	Parents were provided with instructions to download the app and the number to enroll via text.
Bright By Text	Universal	Text messaging program that provides information on topics including physical, mental, and social-emotional health from pregnancy through age 8[18].	A 2018 report found that 92% of Bright By Text subscribers felt more confident because of the texts and had high levels of verbal interaction with their babies[19].	Group 1: Universal (when child was 9 months – after Text4Baby was no longer appropriate)	Parents were provided with the number to enroll via text.
Family Centers (Allegheny County Department of Human Services)	Universal	There are 27 Family Centers that serve families with children under 5. Centers focus on enhancing child development and facilitating parent education and support.		Group 1: Universal, Group 2: Targeted/Universal	Parents opted to receive information about family center(s) most convenient to them to self-enroll or a warm referral, which the Clinical Navigator facilitated with the participant and center via tele-conferencing (an information release was signed at study enrollment).
Nurture Program	Universal	Promotes healthy development in children by pairing mothers with experienced, knowledgeable mentors who use text messaging to provide information, support, and encouragement through the child's first three years of life[20].		Group 2: Targeted/Universal (when parent was a mother-figure and child under age 2 to provide time for program participation)	
PlayReadVIP	Universal	Strengths-based parenting program designed to promote positive parent-child relationships through brief video	PlayReadVIP has been found to enhance parent-child early relational health and child	Group 2: Targeted/Universal,	Referral made internally as program

(formerly Video Interaction Project)		recordings of the parent and child reading and/or playing together followed by immediate real time review[21]. PlayReadVIP sessions were led by non-clinical coaches without advanced training (e.g., bachelor's level), and were offered via telehealth or at a convenient location (e.g., pediatric clinic, WIC*, home). Initially designed to be offered in pediatric primary care, PlayReadVIP sessions coincide with well-child visits[22].<br><br>Infant-toddler session format: Discussion of the child's development, presentation of a developmentally appropriate book or toy, 3-minute recorded parent-child interaction, review of the video to highlight and reinforce strengths observed by the coach, and plan of how to continue reading and playing at home[22].<br><br>Preschool-aged session format: Similar but involved the presentation of a book and toy with aligned themes and included brief recordings of a book reading interaction and a play session. Parents were encouraged to discuss character emotions while reading, integrate writing into play, and to make connections between the story and toy[21].	cognitive-language and social-emotional development[21,23,24].	Group 3: Secondary/Tertiary	was delivered by research staff.
Family Check-Up	Secondary/Tertiary	Family-focused preventive home visiting intervention, designed to improve relational health and child socioemotional outcomes via motivational interviewing techniques[25]. FCU is comprised of an initial interview, assessment, and feedback sessions[26], and is designed to support family strengths,	FCU has been found to improve parenting skills[28], maternal depressive symptoms[29], and child school achievement[30], and to reduce child problem behaviors[28].	Group 3: Secondary/Tertiary<br>Group 4: Tertiary	Referral made internally as program was delivered by research staff.

		assess the parent's willingness to change, and help the family identify areas that need attention. The feedback session helps parents identify goals to address family factors of concern and lays the groundwork for engagement in evidence-based parent management training[27]. FCU was offered in-home, via telehealth, or at a convenient location (e.g., pediatric clinic).			
Smart Beginnings **	Secondary/Tertiary	Integration of PlayReadVIP and FCU to support positive parenting through strengths-based feedback to reduce disparities in school readiness for low-income families[31].	A two-site study of SB found enhanced infant-toddler cognitive environments for children, particularly in domains of reading and teaching[31,32], which in turn mediated enhanced language and early literacy at age 4[33]. The study also showed high levels of program engagement[34], as well as potentiation by PlayReadVIP and FCU in engagement in the other program when offered within the SB model[35].	Group 4: Tertiary	Referral made internally as program was delivered by research staff.
Healthy Families America	Secondary	Home visiting program designed to promote child wellness and prevent maltreatment[36]. Home visits occurred weekly and addressed topics such as infant safety and parenting skills. The local Healthy Families America program was delivered by the Allegheny County Health Department to families with children under age 3; infants had to be enrolled prior to 2 weeks of age.	Program effects have been found for improved parent mental health, parenting knowledge, and responsivity[36].	Group 4: Tertiary (if recruited from birthing hospital and infant was less than 10 days old when offered programs to allow time for referral and enrollment)	The Clinical Navigator facilitated online enrollment (an information release was signed at study enrollment).

**Note that SB was offered as an integrated program for participants in Group 4: Tertiary, but participant in Group 3: Secondary/Tertiary may have selected both PlayReadVIP and FCU, which would be functionally equivalent to the Smart Beginnings program.

sTable 3. Qualifying Criteria By Program Group and Parent

	Parent 1 (P1)				Parent 2 (P2)			
**Parent Participants By Group***	**Group 1: Universal** n (% total sample)	**Group 2: Targeted Universal** n (% total sample)	**Group 3: Secondary/ Tertiary** n (% total sample)	**Group 4: Tertiary** n (% total sample)	**Group 1: Universal** n (% total sample)	**Group 2: Targeted Universal** n (% total sample)	**Group 3: Secondary/ Tertiary** n (% total sample)	**Group 4: Tertiary** n (% total sample)
	233 (22.3%)	246 (23.5%)	313 (30.0%)	253 (24.2%)	72 (33.5%)	45 (20.9%)	57 (26.5%)	41 (19.1%)
**Qualifying Factors by Group**		**Group 2: Targeted Universal** n (% total sample)	**Group 3: Secondary/ Tertiary** n (% total sample)	**Group 4: Tertiary** n (% total sample)		**Group 2: Targeted Universal** n (% total sample)	**Group 3: Secondary/ Tertiary** n (% total sample)	**Group 4: Tertiary** n (% total sample)
**Group 2: Targeted Universal**								
Low Income		238 (96.7%)	222 (70.9%)	245 (96.8%)		24 (53.3%)	15 (26.3%)	25 (61.0%)
Teen Parent		9 (4.5%)	12 (4.7%)	13 (6.3%)		0 (0%)	1 (1.8%)	1 (2.4%)
Birth Complications*		0 (0%)	71 (22.7%)	55 (21.7%)		0 (0%)	0 (0%)	0 (0%)
Does not read with child*		21 (8.5%)	29 (9.3%)	23 (9.1%)		7 (15.6%)	6 (10.5%)	1 (2.4%)
Mild Parenting Challenges*		1 (0.4%)	7 (2.2%)	5 (2.0%)		0 (0%)	1 (1.8%)	0 (0%)
**Group 3: Secondary/Tertiary**								
Mental Health Problems		--	255 (81.5%)	166 (65.6%)		--	52 (91.2%)	23 (56.1%)
Moderate Parenting Challenges*		--	48 (15.3%)	31 (12.3%)		1 (2.2%)	6 (10.5%)	3 (7.3%)
Low Social Support		--	26 (8.3%)	21 (8.3%)			3 (5.3%)	1 (2.4%)
Child Injuries*		--	9 (2.9%)	5 (2 %)		--	1 (1.8%)	0 (0 %)
Child Difficultness*		--	44 (14.1%)	36 (14.2%)		2 (4.4%)	3 (5.3%)	3 (7.3%)
**Group 4: Tertiary**								
Recent Homelessness		--	--	116 (45.8%)		--	--	10 (24.4%)
Child Protective Services Involvement		--	--	153 (60.5%)		--	--	17 (41.5%)
History of Incarceration		--	--	56 (22.1%)		--	--	27 (65.9%)
Opioid Use		--	--	27 (10.7%)		--	--	0 (0%)
Child Problem Behavior*		--	16 (5.1%)	14 (5.5%)		--	3 (5.3%)	0 (0%)

*P1 and P2 group scoring is reported for each child when multiple children were enrolled.

sTable 4. Program Selections By Group and Parent

	Parent 1 (P1)				Parent 2 (P2)			
**Program Selection***	**Group 1: Universal n (%)**	**Group 2: Targeted Universal n (%)**	**Group 3: Secondary/ Tertiary n (%)**	**Group 4: Tertiary n (%)**	**Group 1: Universal n (%)**	**Group 2: Targeted Universal n (%)**	**Group 3: Secondary/Tertiary n (%)**	**Group 4: Tertiary n (%)**
Text4Baby	106 (45.5%)	12 (4.9%)	0 (0%)	0 (0%)	34 (47.2%)	3 (6.7%)	1 (1.8%)	0 (0%)
Bright By Text	9 (3.9%)	14 (5.7%)	1 (0.3%)	0 (0%)	2 (2.8%)	2 (4.4%)	0 (0%)	0 (0%)
Family Center	35 (15.0%)	22 (8.9%)	1 (0.1%)	2 (0.2%)	10 (13.9%)	6 (13.3%)	0 (0%)	0 (0%)
Nurture Program	48 (20.6%)	77 (31.3%)	1 (0.3%)	1 (0.4%)	0 (0%)	1 (2.2%)	0 (0%)	0 (0%)
PlayReadVIP	16 (6.9%)	111 (45.1%)	179 (57.2%)	7 (2.8%)	8 (11.1%)	19 (42.2%)	27 (47.4%)	1 (2.4%)
Family Check-Up	8 (3.4%)	5 (2.0%)	156 (49.8%)	24 (9.5%)	9 (12.5%)	3 (6.7%)	25 (43.9%)	5 (12.2%)
Smart Beginnings	3 (1.3%)	5 (2.0%)	4 (1.3%)	178 (70.4%)	0 (2.2%)	5 (11.1%)	4 (7.0%)	25 (61.0%)
Healthy Families America	0 (0%)	0 (0%)	0 (0%)	3 (1.2%)	0 (0%)	0 (0%)	0 (0%)	1 (2.4%)
**Declined Programs**	37 (16.1%)	36 (14.8%)	27 (8.7%)	23 (9.1%)	18 (25.0%)	6 (13.3%)	7 (12.3%)	12.2 (0%)
**Unable to Contact Parent**	29 (12.6%)	22 (9.0%)	24 (7.7%)	15 (5.9%)	3 (4.2%)	3 (6.7%)	5 (8.8%)	3 (7.3%)

*Notes: Participants could select to participate in multiple programs; families with enrolled P2s were presented with program options for the highest group between P1 and P2; participants rarely requested a less intensive program if they were not satisfied with their options.

Data Statement

De-identified data will be made public at the conclusion of the study. The data will be available indefinitely.

Supplemental sTable 2 references

16. Text4baby. Text4baby. Accessed November 7, 2023. https://www.text4baby.org/

17. Evans WD, Wallace JL, Snider J. Pilot evaluation of the text4baby mobile health program. *BMC public health*. 2012;12(1):1-10.

18. Bright by Text. Bright by Text. Accessed November 7, 2023. https://brightbytext.org/

19. Germeroth C, Kelleman B, Bopp L, Joyce J, Underwood K, Serdiouk M. Bright by Text Evaluation Report. Marzano Research. https://www. brightbytext. org/userfiles/2085/files/BBT …; 2018.

20. Nurture PA. Nurture Program. Nurture PA. Accessed November 7, 2023. https://www.nurturepa.org/

21. Mendelsohn AL, Cates CB, Weisleder A, et al. Reading aloud, play, and social-emotional development. *Pediatrics*. 2018;141(5).

22. Mendelsohn AL, Dreyer BP, Flynn V, et al. Use of videotaped interactions during pediatric well-child care to promote child development: a randomized, controlled trial. Journal of developmental and behavioral pediatrics: *JDBP*. 2005;26(1):34.

23. Mendelsohn AL, Valdez PT, Flynn V, et al. Use of videotaped interactions during pediatric well-child care: impact at 33 months on parenting and on child development. *J Dev Behav Pediatr*. Jun 2007;28(3):206-12. doi:10.1097/DBP.0b013e3180324d87.

24. Cates CB, Weisleder A, Berkule Johnson S, et al. Enhancing Parent Talk, Reading, and Play in Primary Care: Sustained Impacts of the Video Interaction Project. *J Pediatr*. Aug 2018;199:49-56.e1. doi:10.1016/j.jpeds.2018.03.002.

25. Shaw DS, Dishion TJ, Supplee L, Gardner F, Arnds K. Randomized trial of a family-centered approach to the prevention of early conduct problems: 2-year effects of the family check-up in early childhood. *J Consult Clin Psychol*. Feb 2006;74(1):1-9. doi:10.1037/0022-006x.74.1.1.

26. Dishion TJ, Stormshak EA. Intervening in children's lives: An ecological, family-centered approach to mental health care. *American Psychological Association*; 2007.

27. Dishion TJ, Stormshak EA, Kavanagh KA. Everyday parenting: A professional's guide to building family management skills. *Research Press*; 2012.

28. Dishion TJ, Shaw D, Connell A, Gardner F, Weaver C, Wilson M. The Family Check-Up With High-Risk Indigent Families: Preventing Problem Behavior by Increasing Parents' Positive Behavior Support in Early Childhood. *Child Development*. 2008;79(5):1395-1414. doi:10.1111/j.1467-8624.2008.01195.x.

29. Shaw DS, Connell A, Dishion TJ, Wilson MN, Gardner F. Improvements in maternal depression as a mediator of intervention effects on early childhood problem behavior. *Development and psychopathology*. 2009;21(2):417-439.

30. Brennan LM, Shelleby EC, Shaw DS, Gardner F, Dishion TJ, Wilson M. Indirect effects of the family check-up on school-age academic achievement through improvements in parenting in early childhood. *Journal of Educational Psychology*. 2013;105(3):762-773. doi:10.1037/a0032096.

31. Roby E, Miller EB, Shaw DS, et al. Improving parent-child interactions in pediatric health care: A two-site randomized controlled trial. *Pediatrics*. 2021;147(3)

32. Miller EB, Roby E, Zhang Y, et al. Promoting Cognitive Stimulation in Parents Across Infancy and Toddlerhood: A Randomized Clinical Trial. *J Pediatr*. Apr 2023;255:159-165.e4. doi:10.1016/j.jpeds.2022.11.013.

33. Miller EB, Canfield, C. F., Roby, E., Wippick, H., Shaw, D. S., Mendelsohn, A. L., & Morris-Perez, P. A. Enhancing early language and literacy skills for racial/ethnic minority children with low incomes through a randomized clinical trial: The mediating role of cognitively stimulating parent-child interactions. *Child Development*. Dec 2023;00:1-14.

34. Miller EB, Canfield CF, Morris PA, Shaw DS, Cates CB, Mendelsohn AL. Sociodemographic and Psychosocial Predictors of VIP Attendance in Smart Beginnings Through 6 Months: Effectively Targeting At-Risk Mothers in Early Visits. *Prev Sci*. Jan 2020;21(1):120-130. doi:10.1007/s11121-019-01044-y.

35. Canfield CF, Miller EB, Zhang Y, et al. Tiered universal and targeted early childhood interventions: Enhancing attendance across families with varying needs. *Early Childhood Research Quarterly*. 2023/04/01/ 2023;63:362-369. doi:https://doi.org/10.1016/j.ecresq.2023.01.004.

36. Daro DA, Harding KA. Healthy Families America: Using Research to Enhance Practice. *The Future of Children*. 1999;9(1):152-176. doi:10.2307/1602726.
